# Molecular mechanisms of the influenza fusion peptide: insights from experimental and simulation studies

**DOI:** 10.1002/2211-5463.13323

**Published:** 2021-11-08

**Authors:** Diana Lousa, Cláudio M. Soares

**Affiliations:** ^1^ ITQB NOVA Instituto de Tecnologia Química e Biológica António Xavier Universidade Nova de Lisboa Oeiras Portugal

**Keywords:** biophysical assays, hemagglutinin, influenza, membrane fusion, molecular dynamics simulation, virus

## Abstract

A key step in infections by enveloped viruses, such as influenza, is the fusion between the viral envelope and the host cell membrane, which allows the virus to insert its genetic material into the host cell and replicate. The influenza virus fusion process is promoted by hemagglutinin (HA), a glycoprotein that contains three identical monomers composed of two polypeptide chains (HA1 and HA2). Early studies on this protein revealed that HA‐mediated fusion involves the insertion of the HA2 N‐terminal segment into the host membrane and that this segment, known as the fusion peptide, is a key player in the fusion process. This mini‐review highlights the main findings that have been obtained by experimental and computational studies on the HA fusion peptide, which give us a glimpse of its mode of action.

## Graphical abstract

A key step in influenza infection is the fusion of the viral and host membranes, promoted by the protein hemagglutinin, which contains a fusion peptide that inserts and destabilizes the host membrane. This review highlights the major findings of experimental and computational studies, which analyzed the properties of this peptide, including the role of key residues for its fusogenic activity.

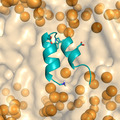

**Abbreviations**
DMPC: 1,2‐dimyristoyl‐sn‐glycero‐3‐phosphocholine
DOPC: 1,2‐dioleoyl‐sn‐glycero‐3‐phosphocholine
DOPE: 1,2‐dioleoyl‐sn‐glycero‐3‐phosphoethanolamine
DPC: dodecylphosphorylcholine
FP: fusion peptide
HA: hemagglutinin
HA1: subunit 1 of hemagglutinin
HA2: subunit 2 of hemagglutinin
IFP: influenza fusion peptide
MD: molecular dynamics
SM: sphingomyelin

Influenza virus causes over 100 000 deaths every year, which rise to millions in pandemic years. Although vaccines are updated early, their efficacy and population coverage are lower than desired. Effective treatments for the disease are scarce and current therapies mainly treat symptoms. Other therapeutic alternatives are clearly needed, but progress has been slow. One interesting possibility is to inactivate the fusion of the viral and host membranes, since this process is crucial for viral infection.

The influenza fusion process is promoted by the surface protein hemagglutinin (HA), which is a class I fusion protein. This structural class is characterized by a homotrimeric arrangement, where each monomer is expressed as single‐chain precursor that requires proteolytic cleavage to make it fusogenic [1]. In the case of HA, cleavage splits the precursor HA0 into two‐disulfide bound chains: HA1 and HA2 [2]. HA1 binds to the host cell receptors, inducing viral uptake by endocytosis, followed by a massive structural change triggered by the acidic endosome pH, during which HA2 becomes extended and inserts the N‐terminal region fusion peptide (FP) into the host membrane [3] (Fig. 1A,B). The protein then refolds, bringing the host and viral membrane into proximity (Fig. 1C). The outer leaflets of the viral and host membrane fuse, forming a hemifusion stalk (Fig. 1D) and, finally, a fusion pore opens (Fig. 1E) [1, 4, 5].

**Fig. 1:**
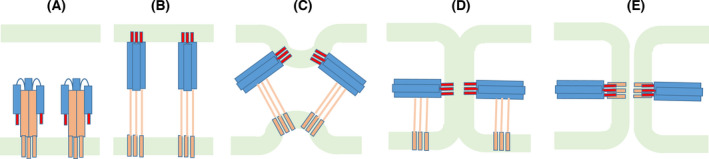
General mechanism of membrane fusion catalyzed by hemagglutinin. The scheme shows the sequence of events that occur during the fusion process. (A) Initially, the protein is in a prefusion conformation. (B) A pH decrease promotes large conformational changes, leading to the formation of an extended intermediate, which enables the insertion of the FP (shown in red) in the host membrane. (C) The protein folds back, zipping up the outer regions against the inner core and pushing the host membrane toward the viral membrane. (D) The two membranes come into contact, forming a hemifusion stalk. (E) The formation of a pore completes the fusion process. Adapted from ref. [3].

The influenza fusion peptide (IFP) is a key player in the fusion process, since it inserts and destabilizes lipid vesicles, inducing hemifusion (fusion of the outer leaflets of two membranes) even in the absence of the rest of the protein [6, 7, 8, 9]. The IFP alone cannot induce complete fusion, since this requires the action of other regions of HA, including the transmembrane domain [10]. It has been shown that mutations within the FP region of HA can either maintain its ability to induce complete fusion, completely abolish its fusogenic ability or result in a protein which can only promote hemifusion, depending on the residue which is mutated [11]. Given the importance of this peptide, several experimental and computational studies have focused on characterizing its structure and effect in model membranes, as well as the role of key residues and external factors such as pH and membrane composition. This mini‐review highlights some of the most relevant findings in this field.

## Defining the fusion peptide

The fusion peptide (FP) can be defined as the segment of the fusion protein that enters the host membrane and promotes fusion. One important aspect of viral FPs is that they can induce lipid mixing of lipid vesicles even when studied on their own, that is, in the absence of the rest of the fusion protein [6, 7]. Another common characteristic is that they tend to be conserved within a virus family [7, 12]. They also tend to be moderately hydrophobic, have a high Ala and/or Gly content, and contain aromatic residues [12]. These and other features can be explored by applying machine‐learning methods to identify viral FP, using packages such as ProPythia [13], which has been tested in similar problems.

The influenza FP is arguably the most thoroughly studied fusion peptide and has become an archetype for class I viral fusion proteins, where the FPs usually correspond to the N‐terminal segment of the fusogenic subunits. The initial suggestion that HA‐mediated fusion would involve the insertion of the N‐terminal segment into the host membrane was based on sequence [14] and structural [15] analyses of HA, followed by the evidence that HA2 interacts with lipids [16] and inserts in the membrane before fusion occurs [17]. Using hydrophobic photolabeling experiments, Brunner and co‐workers pinpointed that the region that inserts into the membrane corresponds to the first 21/22 residues of HA2 [18, 19]. The first evidence that the IFP can induce lipid mixing of membrane vesicles even in the absence of the rest of HA was provided by studies using a 20‐residue long synthetic peptide with the IFP sequence, which showed that this peptide promotes fusion of vesicles at acidic but not at neutral pH [20]. Subsequent studies confirmed the fusogenic activity of the isolated IFP [21, 22].

One relevant question is to determine the actual boundaries of the FP. In the case of influenza, the starting residue corresponds to the first residue of HA2, but determining where it ends is less trivial [6, 8]. One possibility is to consider that the FP is the region that actually inserts into the host membrane. Another way to define the FP is to look at sequence conservation within a family and define it as the set of contiguous conserved residues [6, 9]. One can also adopt a functional perspective and define the fusion peptide as the set of residues that are important to promote lipid mixing of model vesicles [23, 24]. Given the subjectivity inherent to these approaches and criteria used to define the fusion peptide boundaries, we should be aware that these boundaries are somewhat artificial and should not be regarded as intrinsic limits. For this reason, different studies have focused on peptides of different lengths and care must be taken when comparing their results.

## FP structure and orientation in the membrane

**Fig. 2:**
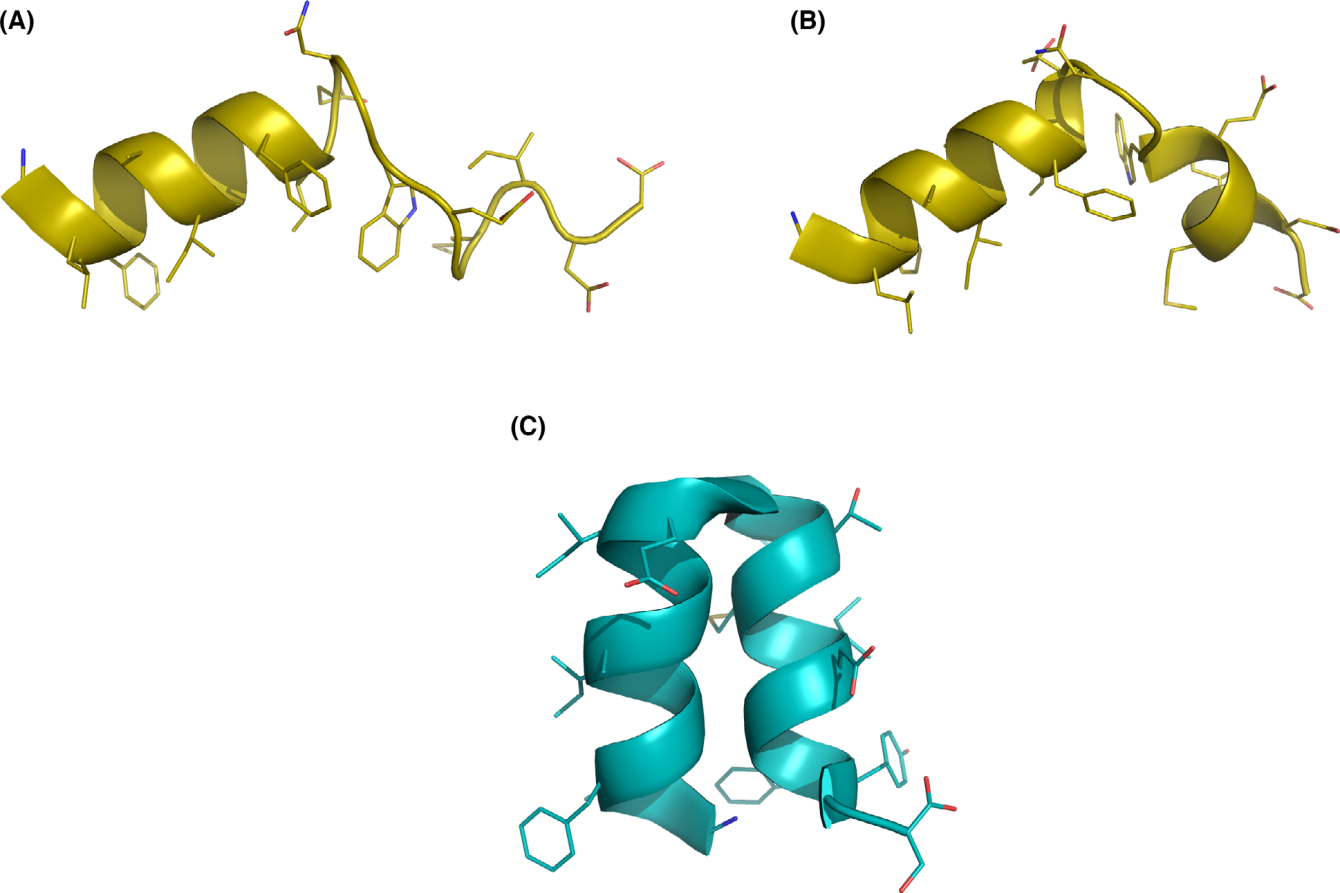
NMR structures of the IFP in detergent micelles. (A) Structure of a synthetic peptide composed by the first 20‐aa residues of HA2 (strain X:31), obtained at pH 7.4 in DPC micelles, determined by ^1^H‐NMR (PDB ID: 1IBN [25]). (B) Structure of the same peptide described in (A), at pH 5 (PDB ID: 1IBO [25]). (C) Structure of a synthetic peptide composed by the first 23‐aa residues of HA2 (from the H1 sero‐subtype) obtained at pH 7.4 in DPC micelles, determined by ^1^H‐NMR (PDB ID: 2KXA [26]). The structure of the same peptide at pH 4 does not have considerable structural changes relative to the structure at pH 7.4. The figures were built with pymol [67], using a cartoon representation for the peptide backbone with carbons colored in gold in the 20‐residue long peptide and in teal in the 23‐residue long peptide.

The first detailed molecular characterization of the IFP structure in a lipid environment was performed by Tamm's laboratory in 2001 [25]. Since this peptide has a high hydrophobic content, the authors designed constructs in which a hydrophilic tail was added to the peptide's C terminus [24]. This allowed them to solubilize the peptides and compare peptides of different lengths (8, 13, 16, and 20 aa residues), showing that the affinity for lipid vesicles and the ability to induce lipid mixing increases with the peptide length and that the IFP has a large helical content when inserted in lipid bilayers [24]. Later on, they used the same construct to obtain the NMR structure of this peptide, which revealed that it adopts a helical inverted V structure in detergent micelles at pH 5 (Fig. 2B), with the C‐terminal helix becoming disordered at pH 7.4 (Fig. 2A) [25]. One important aspect of these studies is that they considered that the FP is composed of the first 20 aa residues of HA2 only, which had been shown to insert into the host cell. Given that residues 21–23 are strictly conserved among HA subtypes and that mutations in W21 and Y22 have been shown to result in negative fusion phenotypes [6], Lorieau et al. [26] analyzed their effect on the structure. This revealed that the 23‐residue long IFP adopts a closed helical‐hairpin structure at pH 7.4 (Fig. 2C), which is not substantially altered at pH 4. At neutral values of pH, the peptide also samples more open conformations [26, 27]. This study also showed that the peptide length affects the packing between the two helices [23]. The closed helical hairpin is stabilized by the presence of four glycines at positions 4, 8, 16, and 20 that form a glycine zipper and by polar interactions between the N‐ and C‐terminal residues, indicating that the last three residues are important to maintain this arrangement [28]. Based on hydrogen exchange rate profile and paramagnetic relaxation enhancement measurements, these authors proposed that the peptide lays on the micelle–water interface with the hydrophobic side exposed to the micelle environment and the more hydrophilic face turned to water [26, 29]. Solid‐state NMR of the 20‐residue long IFP in lipid vesicles (that more realistically represent its interaction with the host membrane) indicated that it adopted a mixture of closed and semi‐closed helix‐turn‐helix structures [30, 31].

**Fig. 3:**
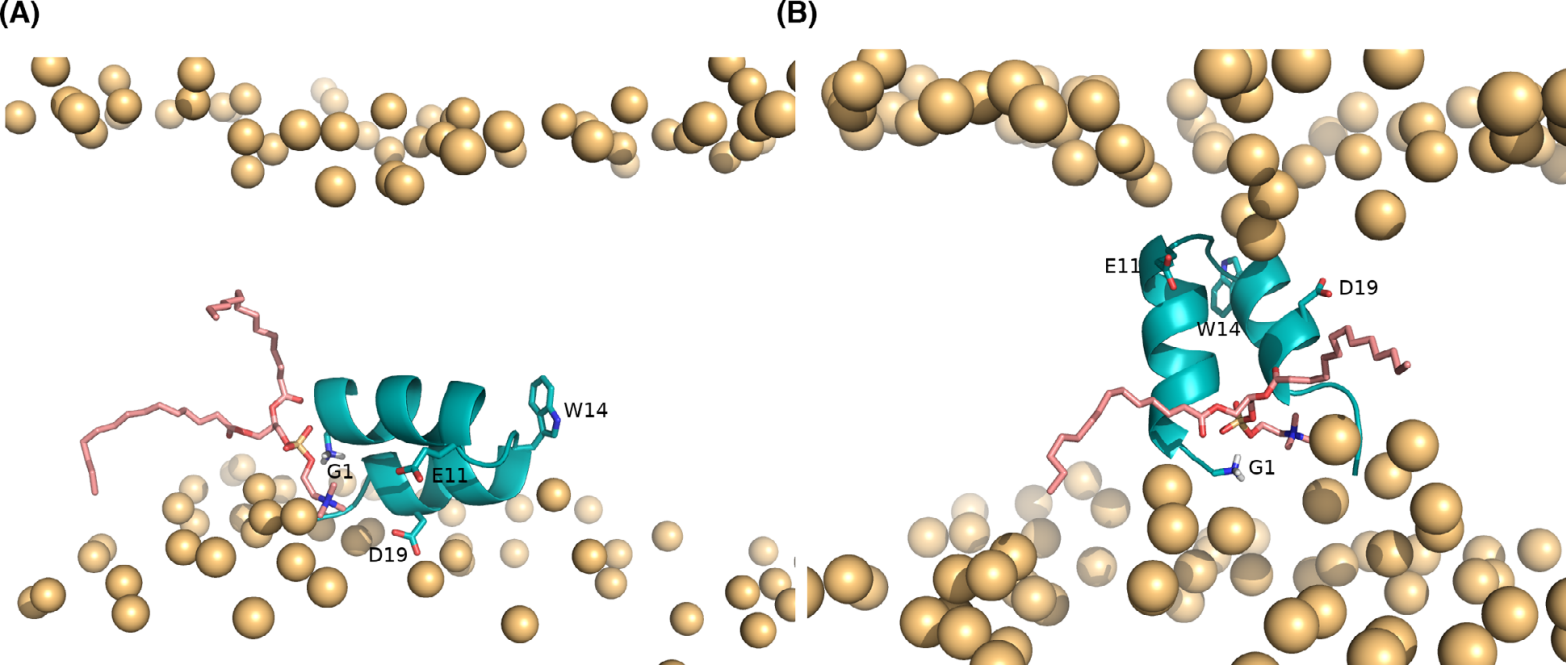
Conformations adopted by the IFP and their effect in the membrane. The conformations were obtained in constant‐pH MD simulations [66] starting from two distinct conformations (labeled as horizontal and vertical) obtained using a self‐assembly approach [37]. (A) Illustration of a lipid tail protrusion event promoted by interaction of a lipid with the peptide N terminus, observed in the constant‐pH MD simulations performed at pH 5 starting from the horizontal conformation [66] (this snapshot corresponds to the 114th ns of replicate 4). (B) Illustration of a lipid tail protrusion event promoted by interaction of a lipid with the peptide N terminus, observed in the constant‐pH MD simulations performed at pH 5 starting from the vertical conformation [66] (this snapshot corresponds to the 597th ns of replicate 4). The images were built with pymol [67]. The IFP is shown using a cartoon representation colored in teal, the lipid phosphorus atoms are depicted by orange spheres and the N terminus of G1, as well as the side chains of E11, W14, and D19 are highlighted using sticks.

Molecular dynamics (MD) simulations have been used to investigate the IFP structure and orientation in lipid membranes, using one of the available NMR structures [32, 33, 34, 35, 36, 37, 38, 39, 40, 41] as a starting point. In some of these studies, the structure remained stable during the simulations [32, 33, 34], whereas in others the peptide could alternate between kinked and extended helical structures [35, 39, 40]. Many of these studies placed the IFP at the lipid head group/lipid tail interface as suggested by the NMR studies and the peptide remained in this region during the simulations [32, 33, 35, 36]. However, this may represent a metastable state, since in the limited simulation time used the peptide is not able to explore states which are very different from the starting one due to the high energy barriers that must be overcome during this transition. To mitigate this limitation, we applied a self‐assembly strategy in which the lipids and water molecules started from a random distribution in the simulation box and were allowed to spontaneously assemble around the peptide [37]. The NMR structure of the 23‐residue long peptide [26] was used in this study, and we observed that the peptide, in 4 out of the 5 replicates where the membrane correctly assembled, adopted a membrane‐spanning conformation with the N‐ and C‐terminal residues interacting with the head groups in one leaflet and the turn residues interacting with the opposing head groups (Fig. 3B). In the remaining replicate, the peptide laid parallel to the membrane plane, at the interface between the lipid head groups and lipid tails of one of the leaflets (Fig. 3A). The helix‐turn‐helix structure was maintained in all of the replicates. This study showed that the IFP can adopt two different orientations in the membrane and introduced the hypothesis that the membrane‐spanning conformation may be important for the peptide's activity. Replica‐exchange MD simulations by Worch et al. [42, 43] have corroborated the idea that the IFP can adopt these two configurations and indicate that the membrane‐spanning configuration corresponds to the lowest free energy minimum for the 23‐residue long fusion peptide with a charged N terminus.

We performed metadynamics simulations to provide a detailed characterization of the conformational energy landscape of the IFP in aqueous solution and in a model membrane composed of DMPC lipids [38]. This enhanced sampling method enables the system to escape free energy minima and extensively explore the conformational space [44]. This showed that in water the peptide is mainly in a random coil state, although it can adopt partially helical structures, which can facilitate its insertion into the membrane. In the membrane, the lowest free energy minimum corresponds to a helix‐turn‐helix structure, very similar to the one obtained in DPC micelles, and this minimum is very stable.

## Mode of action of the IFP

The relevance of viral fusion peptides in membrane fusion has been acknowledged for a long time. However, the exact mechanism by which they promote fusion is still debated and it is arguably the most relevant question in this field. Simulation studies, particularly when combined with experimental analysis, have provided important insights into this subject.

Proposed mechanisms, based on coarse‐grained MD simulations, involve altering membrane curvature leading to the formation of cubic lipid phases [45, 46] or forming peptide bundles with a central pore, which drives the elongation of the fusion stalk [47]. Other possible mechanisms involve lipid ordering and increasing the rigidity of specific membrane regions. Using electron spin resonance experiments in the presence of DMPC vesicles, Freed and co‐workers found that the IFP rigidifies the head group region, while having little effect on the lipid tails [48]. Other groups have observed that the IFP enhances the order of the lipids, particularly in the interfacial region, using fluorescence anisotropy measurements of the IFP in membranes composed of DOPC/DOPE/SM/cholesterol [49]. On the other hand, several MD simulation studies suggest that the IFP decreases the order parameters of the lipids that interact with the peptide [32, 33, 36, 37, 50, 51]. Overall, the effect of IFP (and other viral FPs) on membrane order and rigidity seems to be rather complex and dependent on membrane composition, peptide length, and concentration, varying with membrane depth (for a review, see [52]).

An important phenomenon that is thought to play a role in FP‐induced membrane fusion is lipid tail protrusion, which occurs when a lipid tail protrudes beyond the corresponding phosphate group and was first observed in coarse‐grain simulations [53] and, shortly after, in large‐scale atomistic simulations [54]. Kasson et al. [54] simulated the fusion process between two lipid vesicles and found that the transition state of this process is defined by the contact of a few lipid tails from the proximal leaflets of the fusing vesicles, which occurs when lipid tails protrude into the hydrophilic region. They observed that the probability of lipid tail protrusion increases significantly in the presence of the IFP and hypothesized that this is one of the key mechanisms by which this peptide promotes fusion [54, 55]. Similar observations were made in a study performed in our laboratory, where we used a self‐assembly approach to study the IFP in the presence of a spontaneously assembled DMPC [37]. Interestingly, we observed that the probability of lipid tail protrusion is considerably higher when the peptide adopts a membrane‐spanning conformation, which indicates that this state is important for fusion [37].

Recently, the Kasson's laboratory provided novel insights into this subject by simulating the fusion process of a proteoliposome (mimicking the virus envelope) and a planar lipid bilayer (representing the host membrane) [56]. They simulated three copies of the complete HA2, with the transmembrane domain inserted in the liposome and the IFP on the planar membrane, and assessed how they promote fusion, by combining atomistic and coarse‐grained MD to model different stages of the process. This study indicates that fusion is a two‐stage process: First, the IFP promotes lipid tail protrusion, which drives stalk formation, and then, the IFP perturbs the distal leaflets and helps to form the hemifusion diaphragm, which finally results in the opening of a fusion pore [56]. The effect of the IFP on the distal leaflets requires a deep insertion into the membrane, showing once again the importance of membrane‐spanning conformations [56], similar to the one that was first proposed by us [37].

The IFP ability to exchange between superficial and membrane‐spanning configurations has also been shown by Worch et al. [57], who performed extensive MD simulations, including temperature replica exchange and potential of mean force calculations, combined with experiments, to characterize the peptide's conformational landscape. This study also confirmed that the membrane‐spanning configuration allows the peptide to strongly perturb the membrane and considerably increase the extent of lipid tail protrusion relative to a peptide‐free membrane.

## The role of key residues and their protonation

To have a detailed understanding of how the IFP promotes membrane fusion, we need to pinpoint the role of key residues in this process and determine how the endosome pH affects their protonation state and effect on the host membrane. Experimental studies have tried to identify key IFP residues by introducing point mutations and determining how they affect fusion, both in the context of the complete HA [38, 58, 59] as well as using the isolated FP to perform lipid mixing assays [21, 60, 61, 62] (for a review, see [6]). These studies point to the importance of the first N‐terminal residues, particularly G1, for membrane fusion [6, 59, 60, 61, 63, 64]. The aromatic residues W14, W21, and Y22 are also important for the peptide's activity [6, 65], whereas the acidic residue E11 affects the pH dependence of fusion [6, 58, 62]. Using a combination of simulations and experiments, Worch et al. [57] revealed that W14 has a crucial role in the IFP transition from the membrane surface to the membrane‐spanning conformation, by stabilizing the tight helical‐hairpin structure. They also showed that the protonation state of residue E11, as well as its mutation to alanine, affects the depth of peptide insertion in the membrane.

The position of glycine residues within the IFP sequence also seems to be important for its activity, which is likely due to their role in stabilizing the tight helical‐hairpin structure. In fact, an extensive simulation analysis applying metadynamics simulations, performed in our laboratory, showed that the G4A/G8A/G16A/G20A mutant has an unstable structure in a DMPC membrane [38]. In this study, which combined simulation and spectroscopic analyses, we also tested the G1V, W14A, and G12A/G13A mutations and found that they did not have a considerable impact on the peptide's structure. These mutations did, however, affect the peptide's ability to induce lipid mixing, mainly due to their reduced affinity for lipidic environments, which results in a lower peptide concentration inside the membrane [38]. This indicates that the peptide/lipid ratio is important for fusion, which has also been shown by others [9, 22, 60].

Several simulation studies show that the N‐terminal end of the peptide is a key player in the fusion process, in accordance with the previously described mutation analyses. The simulations show that the N‐terminal G1 interacts strongly with the phosphate groups, which results in head group intrusion and lipid tail protrusion (an illustration of this effect is shown in Fig. 2) [36, 37, 38, 66]. Interestingly, both simulation and experimental studies have shown that having a free NH_3_^+^ terminal is crucial for the IFP effect on the membrane [20, 42, 56, 66]. Using constant‐pH MD simulations, we have recently shown that pH plays a crucial role in the IFP interaction with the membrane: At the endosome pH, the N‐terminal is predominantly protonated and frequently interacts with the lipid ester groups [66]. This study also showed that, by controlling the protonation state of ionizable groups, pH affects the orientation of the IFP in the membrane: the membrane‐spanning conformation (proposed to be important for membrane fusion) is considerably more stable at pH 5 than at pH 7. This is in line with fluorescence resonance energy transfer data showing that the peptide's ability to induce lipid mixing is twofold higher at low pH [66].

## Concluding remarks

A large body of experimental and computational studies have analyzed the IFP, which is a key player in the influenza fusion process. The picture that emerges from these studies shows that the IFP adopts a helix‐turn‐helix structure in the membrane, with the arrangement between the two helices being affected by the peptide length. Although the IFP was initially proposed to lay at the head group–lipid tail interface, simulation studies suggest that it can adopt different arrangements, including membrane‐spanning and interfacial conformations and that these arrangements are affected by different factors, such as the protonation state.

The mode of action of this peptide is thought to involve a combination of different mechanisms, including altering membrane curvature, perturbing lipid order, and inducing lipid tail protrusion and lipid head intrusion. The interaction of key residues, in particular those of the N‐terminal, with the lipid phosphates and ester groups plays an important role in this process, which is influenced by the residue's protonation state. At low pH, the peptide can adopt a membrane‐spanning conformation and interact with the distal leaflet, being more effective in promoting fusion. Factors such as peptide/lipid ratio and membrane composition also affect this process and it is becoming clear that several peptides act in concert to destabilize the host membrane [9].

In spite of this large body of knowledge, further research is needed to answer open questions, such as determining the most relevant effect of the IFP for membrane fusion, validating *in vitro* findings in a biological context and assessing whether other viral FP use similar mechanisms.

## Conflict of interest

The authors declare no conflict of interest.

## Author contributions

DL and CMS defined the scope of this review, the topics that would be covered and the perspective that would be adopted; DL and CMS reviewed the literature; DL and CMS wrote and reviewed the manuscript.

## Data Availability

This review article does not present any novel data.
